# The Use of “Antigens” in Diagnosis by the Wassermann Reaction: The Necessity for Further Investigation

**Published:** 1919

**Authors:** 


**The Use of " Antigens " in Diagnosis by the Wassermann Reaction : The Necessity for Further Investigation.** By C. H. Browning, M. D., and E. L. Kenneway, M. D., *The Lancet*, November 30, 1918.

While appreciating the value of adopting a " standard " antigen in making the Wassermann reaction, the authors point out the limitations of those in common use, which should preclude their adoption as " standard ".

The property of a syphilitic antigen is that when mixed with syphilitic serum (heated to 56° C) in suitable proportion and added to fresh guinea-pig serum, it deprives the serum of its hemolytic power.

Attention is called to the evolution of knowledge as to the syphilitic antigen. It is desirable that the quantitive difference between a negative and a positive serum should be as great as possible, as measured by the excess of the amount of complement which the serum-antigen will inactivate, because this is the only index of positiveness which exists. Examination shows that alcoholic heart extracts possess this [quality to an outstanding degree, therefore human heart extract has been employed as syphilitic antigen for routine purposes. Other workers have used a similar extract with the addition of cholesterol. Some laboratory workers found that the heart " lipoids " plus cholesterol and certain normal sera led to a great inactivation of complement. They continued to use liver-lecithin, feeling that the smaller complement fixation was compensated by the fact that in a series used with a parallel series plus cholesterol, if a given serum caused more complement inactivation, it was an indication of its syphilitic character.

The crude heart extracts have a tendency to cause considerable complement fixation with non-syphilitic sera. On the other hand, such an antigen as that first advocated by McIntosh and Fildes has found much favor. Its popularity is evidenced by the fact that of the four methods of performing the test recommended by the Special Committee from the Medical Research Committee, three employ this antigen. The Committee states : " Of the various extracts which have been recommended at one time or another, that prepared from heart and reinforced with cholesterol, as recommended by McIntosh and Fildes, will be found simple to prepare and as satisfactory as any in use at the present moment ".

Walker and Swift from results of tests believe that the antigenic property of cholesterol-heart extracts is especially evident when testing sera from recently treated cases of syphilis.

The authors have carried out a series of parallel tests employing liver-lecithin plus cholesterol, and McIntosh and Fildes' heart extract plus cholesterol. The results confirm their earlier observation that liver-lecithin plus cholesterol antigen with many positive sera gives a smaller amount of complement fixation than other antigens; the liver-lecithin-cholesterol antigen leads to greater complement fixation than does the heart-cholesterol antigen.

Not only may one antigen differ from another as regards optimum concentration, but the amount of serum necessary to elicit maximum complement fixation may vary.

Results have led to adoption of the following routine in Wassermann tests : For each serum, two series of three tubes are set up : (*a*) containing liver-lecithin-cholesterol (1 : 8 turbid emulsion) 0.3 cc. plus 0.025 cc. patient's heated serum ; and (*b*) heart extract plus cholesterol (1 : 30 rapidly mixed emulsion) 0.5 cc. plus 0.05 cc. patient's heated serum along with increasing amounts of complement in each series.

Clearing up the present uncertainty is urged by concentration on study of (1) cases of syphilis with previous positive reaction and present reaction nearly extinguished ; (2) untreated cases from congenital syphilitic families; and (3) cases where suspicious or positive reactions have been obtained.

For certainty in diagnosis it is advisable always to use both antigens. If only one is used, all sera should be preserved by freezing that the test may be repeated with the other antigen.

